# Advances in the Diagnosis of Barrett’s Esophagus

**DOI:** 10.3390/diagnostics16182898

**Published:** 2026-09-09

**Authors:** Ravi Patel, Ali Ghazanfar, Rida Fatima, Rushin Shah, Aman Patel, Vikash K. Karmani, Devanshi Bhatt, Muhammad Bilal, Zarak H. Khan, Haider Ghazanfar

**Affiliations:** 1 Trinity Health Oakland, Pontiac, MI 48341, USA; 2 BronxCare Health System, New York, NY 10457, USA; 3 Department of Internal Medicine, East Carolina University, Greenville, NC 27858, USA; 4 Department of Internal Medicine, University of Connecticut, Storrs, CT 06269, USA; 5 Banner Del E Webb Medical Center, Sun City West, AZ 85375, USA; 6 Department of Internal Medicine, Jinnah Sindh Medical University, Karachi 75510, Pakistan; vikashkarmani@gmail.com; 7 Department of Gastroenterology, East Carolina University, Greenville, NC 27858, USA; 8 Department of Gastroenterology, Virginia Tech Carilion School of Medicine, Roanoke, VA 24016, USA

**Keywords:** Barrett’s esophagus, esophageal adenocarcinoma, dysplasia, endoscopic imaging, narrow-band imaging, biomarkers, Cytosponge, liquid biopsy, artificial intelligence, cancer screening

## Abstract

Barrett’s esophagus (BE), the intestinal metaplasia arising from chronic gastroesophageal reflux disease, is the principal identifiable precursor of esophageal adenocarcinoma (EAC), whose incidence rose from 0.4 to 2.8/100,000 person-years between 1975 and 2017 and whose prognosis, once symptomatic, remains poor. Because outcomes depend on intercepting the metaplasia–dysplasia–carcinoma sequence, diagnostic accuracy is decisive. White-light endoscopy with Seattle-protocol biopsy remains the reference standard, yet it is constrained by the following three interrelated weaknesses: sampling error, as random forceps biopsies interrogate only about 3.5% of the Barrett’s mucosa; poor reproducibility of dysplasia grading, with interobserver agreement of only κ 0.24–0.27 for the pivotal distinction of low-grade dysplasia; and a substantial burden of missed disease, with roughly one-quarter of EACs diagnosed within a year of an index endoscopy reported as nondysplastic. This review synthesizes the technologies converging to address these gaps. Advanced imaging, encompassing high-definition endoscopy, narrow-band imaging, acetic acid chromoendoscopy, and the optical-biopsy platforms confocal laser and volumetric laser endomicroscopy, raises dysplasia yield by approximately 34% over standard white-light examination. Image-enhanced endoscopy improves targeted detection while remaining complementary to structured biopsy sampling. Molecular, genetic, and epigenetic biomarkers, notably DNA-content abnormalities, p53 immunohistochemistry, and multi-gene methylation panels, add an objective read on progression risk. Their pairing with non-endoscopic sampling, including Cytosponge-TFF3, capsule endoscopy, exhaled volatile organic compounds, and circulating microRNA liquid biopsy, is reshaping screening at population scale, while artificial intelligence standardizes interpretation and narrows the expert–nonexpert gap. Together these advances point toward a risk-stratified, multimodal paradigm, though prospective validation and cost-effectiveness evidence remain prerequisites for guideline adoption.

## 1. Introduction

Barrett’s esophagus (BE) is a condition that develops as a complication of chronic gastroesophageal reflux disease (GERD). Long-standing GERD leads to persistent esophageal exposure to gastric acid, resulting in chronic inflammation and mucosal injury. As a result, the squamous epithelium of the esophageal mucosa is replaced with intestinal-type columnar epithelium containing goblet cells [1]. The 2022 American College of Gastroenterology guideline defines BE as the presence of columnar mucosa extending at least 1 cm above the gastroesophageal junction with histology showing intestinal metaplasia characterized by goblet cells [2]. BE affects approximately 5% of people in the US, develops in 5 to 15% of patients with chronic GERD, and is associated with the progression to esophageal adenocarcinoma (EAC) [3,4]. Risk factors for BE include male sex, obesity, age over 50 years, white race, tobacco smoking, and a family history of BE or EAC in a first-degree relative [1]. Each additional risk factor increases the prevalence of BE in patients with GERD by 1.2% [2].

Over time, driven by persistent inflammation, metaplastic cells progress through non-dysplastic Barrett’s, low-grade dysplasia, high-grade dysplasia, and eventually esophageal adenocarcinoma. The annual risk of cancer progression in patients with Barrett’s esophagus is presented in Table 1 [5].

The incidence of EAC has increased from 0.4 per 100,000 person-years in 1975 to 2.8 per 100,000 person-years in 2017 [6]. A study of 8929 BE patients from a healthcare organization in Northern California showed that the risk of esophageal adenocarcinoma was increased by 24-fold [7]. Data from a multicenter U.S. consortium showed that the prevalence of high-grade dysplasia and EAC on index endoscopy increased by 148% and 112%, respectively, over the past 25 years [8]. These findings underscore the importance of timely screening of patients at high risk of developing Barrett’s esophagus and of the accurate, early diagnosis. This review examines how Barrett’s esophagus is diagnosed today, why the conventional approach falls short, and how the emerging technologies are reshaping the field.

## 2. Methodology

### 2.1. Study Design

We performed a narrative review of current and emerging approaches to the diagnosis and risk assessment of Barrett’s esophagus (BE). The review includes conventional white-light endoscopy and histopathology, advanced endoscopic imaging techniques, molecular and epigenetic biomarkers, non-endoscopic and minimally invasive sampling methods, and artificial intelligence (AI)-based approaches.

We selected a narrative review format because the literature in this area covers a wide range of diagnostic technologies and study designs, and our goal was to provide a clinically focused overview of the field rather than answer a single focused question or calculate a pooled estimate of diagnostic accuracy.

The evidence bases therefore drew on diagnostic accuracy studies, randomized controlled trials, observational studies, biomarker studies, cost-effectiveness analyses, systematic reviews and meta-analyses, and current clinical practice guidelines.

### 2.2. Data Sources and Search Strategy

We searched PubMed/MEDLINE for studies published between 1 January 2000 and 31 May 2026. The final search was conducted on 10 June 2026. The search was designed to capture the literature on the diagnosis and risk assessment of BE, including conventional and advanced endoscopic imaging, histopathology, molecular and genetic biomarkers, epigenetic markers, non-endoscopic sampling, minimally invasive diagnostic approaches, and artificial intelligence. The strategy combined Medical Subject Heading (MeSH) terms with title/abstract (tiab) free-text terms across three concept blocks, joined by the Boolean operator AND, with terms within each block joined by OR. The exact reproducible strategy was as follows:

Block 1: Disease and Pathology: ”Barrett Esophagus”[Mesh] OR “Barrett esophagus”[tiab] OR “Barrett’s esophagus”[tiab] OR “Barrett oesophagus”[tiab] OR “Barrett’s oesophagus”[tiab] OR “columnar-lined esophagus”[tiab] OR “Adenocarcinoma”[Mesh] OR “esophageal adenocarcinoma”[tiab] OR “oesophageal adenocarcinoma”[tiab] OR “Esophageal Neoplasms”[Mesh] OR “dysplasia”[tiab] OR “intraepithelial neoplasia”[tiab] OR “intestinal metaplasia”[tiab].

AND

Block 2: Diagnostic/risk-assessment purpose: ”Diagnosis”[Mesh] OR “diagnosis”[tiab] OR “Early Detection of Cancer”[Mesh] OR “screening”[tiab] OR “surveillance”[tiab] OR “Risk Assessment”[Mesh] OR “risk stratification”[tiab] OR “risk assessment”[tiab] OR “disease progression”[tiab].

AND

Block 3: Diagnostic and risk-assessment technologies: ”Endoscopy, Gastrointestinal”[Mesh] OR “endoscopic imaging”[tiab] OR “white-light endoscopy”[tiab] OR “high-definition endoscopy”[tiab] OR “high definition endoscopy”[tiab] OR “Prague classification”[tiab] OR “Seattle protocol”[tiab] OR “four-quadrant biopsy”[tiab] OR “Narrow Band Imaging”[Mesh] OR “narrow-band imaging”[tiab] OR “narrow band imaging”[tiab] OR “chromoendoscopy”[tiab] OR “acetic acid”[tiab] OR “Microscopy, Confocal”[Mesh] OR “confocal laser endomicroscopy”[tiab] OR “Tomography, Optical Coherence”[Mesh] OR “optical coherence tomography”[tiab] OR “volumetric laser endomicroscopy”[tiab] OR “Histology”[tiab] OR “histopathology”[tiab] OR “Biomarkers, Tumor”[Mesh] OR “biomarkers”[tiab] OR “biomarker”[tiab] OR “molecular markers”[tiab] OR “genetic markers”[tiab] OR “Tumor Suppressor Protein p53”[Mesh] OR “p53”[tiab] OR “TP53”[tiab] OR “immunohistochemistry”[tiab] OR “Loss of Heterozygosity”[Mesh] OR “loss of heterozygosity”[tiab] OR “Aneuploidy”[Mesh] OR “aneuploidy”[tiab] OR “tetraploidy”[tiab] OR “DNA ploidy”[tiab] OR “Flow Cytometry”[Mesh] OR “flow cytometry”[tiab] OR “image cytometry”[tiab] OR “In Situ Hybridization, Fluorescence”[Mesh] OR “fluorescence in situ hybridization”[tiab] OR “FISH”[tiab] OR “Epigenesis, Genetic”[Mesh] OR “epigenetic”[tiab] OR “DNA methylation”[tiab] OR “DNA Methylation”[Mesh] OR “promoter hypermethylation”[tiab] OR “methylation panel”[tiab] OR “p16”[tiab] OR “CDKN2A”[tiab] OR “Cytosponge”[tiab] OR “Trefoil Factor-3”[Mesh] OR “trefoil factor 3”[tiab] OR “TFF3”[tiab] OR “non-endoscopic screening”[tiab] OR “non-endoscopic sampling”[tiab] OR “minimally invasive”[tiab] OR “Capsule Endoscopy”[Mesh] OR “capsule endoscopy”[tiab] OR “esophageal capsule”[tiab] OR “PillCam”[tiab] OR “Liquid Biopsy”[Mesh] OR “liquid biopsy”[tiab] OR “circulating tumor DNA”[tiab] OR “cell-free DNA”[tiab] OR “MicroRNAs”[Mesh] OR “microRNA”[tiab] OR “miRNA”[tiab] OR “Breath Tests”[Mesh] OR “breath testing”[tiab] OR “breath analysis”[tiab] OR “volatile organic compounds”[tiab] OR “electronic nose”[tiab] OR “Artificial Intelligence”[Mesh] OR “artificial intelligence”[tiab] OR “machine learning”[tiab] OR “deep learning”[tiab] OR “Neural Networks, Computer”[Mesh] OR “convolutional neural network”[tiab] OR “computer-aided diagnosis”[tiab] OR “computer-aided detection”[tiab].

The following three current society guidelines were incorporated directly into the evidence synthesis: the 2022 American College of Gastroenterology (ACG) guideline on the diagnosis and management of BE [2], the 2023 European Society of Gastrointestinal Endoscopy (ESGE) guideline on the diagnosis and management of BE [9], and the 2025 American Gastroenterological Association (AGA) Clinical Practice Guideline on BE surveillance [10]. Reference lists of relevant articles, reviews, and guidelines were hand-searched for studies missed by the database query, and earlier publications were retained where they supplied foundational evidence for established diagnostic techniques.

### 2.3. Eligibility Criteria

We included peer-reviewed studies that addressed the diagnosis, screening, surveillance, or risk stratification of BE or its progression to esophageal adenocarcinoma. Eligible designs included diagnostic accuracy studies, randomized controlled trials, prospective and retrospective cohort studies, case–control studies, systematic reviews and meta-analyses, biomarker validation studies, cost-effectiveness and decision analyses, and clinical practice guidelines or consensus statements. Studies were excluded if they were not published in English, fell outside the scope of BE diagnosis or risk assessment, or otherwise did not bear on the review’s objectives.

### 2.4. Study Selection and Data Synthesis

Three authors (R.P., Z.H.K., and H.G.) screened all titles and abstracts identified by the search to determine potential eligibility and then reviewed the full text of potentially relevant articles against the eligibility criteria. Disagreements regarding inclusion were resolved through discussion and consensus. Because this was a narrative review, studies were selected based on their relevance to the review topic, quality of the available evidence, and potential clinical applicability.

When several studies addressed the same diagnostic technology, we weighed the evidence by study design to limit redundancy and selective emphasis. Systematic reviews, meta-analyses, and randomized controlled trials carried the most weight. Large prospective and multicenter validation studies came next, followed by smaller prospective studies, with retrospective and single-center studies weighed least. Where designs were comparable, we favored studies with larger samples, more rigorous reference standards, external validation, and more recent publication dates. Current society guidelines serve as the primary sources for consensus recommendations.

The findings were organized according to the major diagnostic approaches covered in the review. The evidence was synthesized qualitatively, with emphasis on the strengths and limitations of each approach, its current clinical role, and areas where additional validation is needed.

### 2.5. Limitations

No formal meta-analysis or pooled statistical analysis was performed. A formal risk of bias assessment was also not performed because the purpose of this article was to provide a narrative clinical synthesis rather than a systematic review with quantitative evidence synthesis.

### 2.6. Ethical Considerations

This review is based entirely on the previously published literature and did not involve new studies on human participants or animals.

## 3. Conventional Diagnostic Methods

### 3.1. Upper Endoscopy (White-Light Endoscopy)

White-light endoscopy (WLE) remains the reference-standard modality for the diagnosis of Barrett’s esophagus (BE). The examination is most often performed by the peroral route under conscious sedation, although unsedated trans-nasal endoscopy is an increasingly studied alternative. The diagnosis rests on recognizing a columnar-lined distal esophagus extending above the gastroesophageal junction (GEJ); accurate identification of the GEJ is the key landmark that distinguishes true BE from intestinal metaplasia of the gastric cardia and from a hiatal hernia [11]. Diagnostic yield is highly dependent on the quality of the inspection: contemporary quality standards recommend cleansing the mucosa and dedicating at least one minute of inspection time per centimeter of the Barrett’s segment, as longer, more careful inspection correlates with higher rates of dysplasia detection [12]. The 2022 ACG guideline recommends high-definition white-light endoscopy for surveillance, while the 2023 ESGE guideline recommends a minimum inspection time of 1 min per centimeter of BE during surveillance, standardized photo documentation, use of the Prague classification, and targeted biopsies of visible abnormalities followed by systematic four-quadrant biopsies [2,9]. The 2025 AGA guideline similarly emphasizes high-quality endoscopic examination and recommends combining high-definition white-light endoscopy with chromoendoscopy during surveillance [10].

The endoscopic extent of BE is reported using the Prague C & M classification, a consensus-derived system that records the circumferential (C) length of metaplasia and the maximal (M) length, the latter including any noncircumferential “tongue-like” projections of columnar epithelium above the GEJ [13]. Standardized measurement improves reporting consistency and helps with risk stratification, biopsy planning, and surveillance intervals. In the original validation exercise by consensus experts, the criteria showed high reliability for segments ≥ 1 cm (reliability coefficients of approximately 0.95 for C and 0.94 for M extent) but performed poorly for segments < 1 cm (coefficient ~0.22), reflecting the difficulty of grading ultra-short segments and of localizing the GEJ precisely [13]. This reliability has since been reproduced among gastroenterology trainees after brief standardized training, with intraclass correlation coefficients of 0.94 for C and 0.96 for M extent, and of 0.92 and 0.90 for recognition of the GEJ and diaphragmatic hiatus, respectively [14].

### 3.2. Histopathologic Confirmation

Endoscopic recognition of BE must be confirmed histologically. Because dysplasia and early neoplasia are frequently focal and variable, guidelines recommend systematic four-quadrant biopsies combined with targeted sampling of any visible mucosal abnormality [15]. The Seattle protocol formalizes this approach, prescribing four-quadrant biopsies at 2 cm intervals in patients without known dysplasia and at 1 cm intervals in those with prior dysplasia, supplemented by targeted biopsies of nodules, ulceration, or other irregularities [15]. Relative to unstructured random sampling, this systematic technique increases the detection of intestinal metaplasia, dysplasia, and early esophageal adenocarcinoma (EAC). This structured sampling strategy is consistent with the 2022 ACG guidelines [2]. The 2025 AGA guideline similarly emphasizes structured biopsy sampling during surveillance and stresses confirmation of BE-related neoplasia by an expert pathologist [10]. 

Biopsy specimens are evaluated with hematoxylin and eosin (H&E) staining. Histopathology grades neoplastic change along the metaplasia–dysplasia–carcinoma sequence. Low-grade dysplasia (LGD) is characterized by nuclear enlargement, hyperchromasia, and increased mitotic activity with relatively preserved glandular architecture and polarity, whereas high-grade dysplasia (HGD) shows marked nuclear atypia and pleomorphism, brisk mitotic activity, and disrupted glandular architecture with loss of polarity [16]. Progression through these stages precedes invasive EAC and forms the biological basis for surveillance.

### 3.3. Limitations of Conventional Diagnostic Methods

Although endoscopy with histopathologic confirmation remains the diagnostic gold standard, its real-world performance is constrained by the following three interrelated limitations: sampling error, diagnostic variability, and missed dysplasia and neoplasia.

#### 3.3.1. Sampling Error

Even meticulous four-quadrant biopsy interrogates only a small fraction of the Barrett’s surface. The average BE segment has an estimated mucosal surface area of roughly 14 cm^2^, of which random forceps biopsies sample only about 0.5 cm^2^, approximately 3.5% of the mucosa [12]. Because dysplasia and early EAC are patchy and often endoscopically invisible, focal lesions are readily missed in the tissue lying between biopsy sites. Poor adherence compounds the problem as follows: the protocol is time-consuming, with compliance falling as segment length increases, and the sensitivity for dysplasia detection vary widely from roughly 28% to 85% [12].

#### 3.3.2. Diagnostic Variability

The histologic diagnosis of dysplasia is subjective and poorly reproducible, chiefly because reactive and regenerative atypia driven by reflux-induced inflammation closely mimics true dysplasia. Interobserver agreement is only fair for the clinically pivotal distinction between no dysplasia and indefinite-for/low-grade dysplasia (kappa ~0.24–0.27), improving to substantial agreement only when separating HGD or adenocarcinoma from lesser grades (kappa ~0.58–0.62) [17]. The consequences are greatest for LGD: in a community-based cohort of 147 patients originally labeled with LGD, expert pathology review downgraded 85% to nondysplastic or indefinite disease, and this reclassification carried major prognostic weight, as histologically confirmed LGD progressed to HGD or cancer at 13.4% per patient-year, compared with only 0.49% per year among down-staged cases [18]. The clinical importance of this variability is reflected in current guidelines. The ACG, ESGE, and AGA all emphasize expert pathology review when dysplasia is suspected, particularly because management decisions depend heavily on accurate distinction between indefinite for dysplasia, low-grade dysplasia, and high-grade dysplasia. This provides an important rationale for developing objective molecular and computational approaches that may complement conventional histopathologic assessment.

#### 3.3.3. Missed Dysplasia and Neoplasia

Sampling error and diagnostic variability, compounded by subtle or overlooked mucosal lesions and operator-dependent examination quality, translate into a substantial burden of missed disease. Meta-analyses estimate that approximately one-quarter of EACs (range ~16–37%) are diagnosed within one year of an index endoscopy reported nondysplastic—the so-called post-endoscopy EAC—implying that the lesion was present but undetected at the initial examination [19,20]. Detection is further influenced by endoscopist experience and by the inherent limitations of standard white-light imaging in resolving early, flat, or subtle Barrett’s-related changes. Collectively, these shortcomings provide the rationale for the advanced imaging and enhanced tissue-sampling technologies discussed in the sections that follow.

## 4. Advances in Endoscopic Imaging

The endoscopic assessment of BE has undergone a paradigm shift over the past two decades, following the recognition that traditional white-light endoscopy (WLE) with random four-quadrant biopsies—the Seattle protocol—is inherently flawed by its susceptibility to sampling error, interobserver differences in pathological interpretation, and inconsistent implementation. These limitations have driven the development and validation of imaging technologies that fall into the following two broad categories: enhanced imaging techniques, which improve visualization of the mucosal surface, and optical biopsy techniques, which provide real-time microscopic analysis of tissue in vivo. In a meta-analysis of advanced imaging modalities, these technologies increased the diagnostic yield for dysplasia or neoplasia by approximately 34% relative to standard WLE [21].

### 4.1. Enhanced Imaging Techniques

#### 4.1.1. High-Definition Endoscopy

High-definition endoscopy (HDE), which uses charge-coupled-device sensors exceeding one million pixels, provides markedly better mucosal resolution than standard-definition systems and is now the minimum accepted standard for BE surveillance under contemporary society guidelines. Although HDE does not characterize tissue itself, its resolution improves recognition of subtle mucosal irregularities, villous pit patterns, and focal nodularity that may harbor dysplasia. In a retrospective surveillance cohort, HDE outperformed standard-definition endoscopy for targeted detection of dysplasia (odds ratio [OR] 3.27; 95% CI, 1.27–8.40) and for overall dysplasia yield on combined random and targeted biopsies (OR 2.36; 95% CI, 1.50–3.72); however, it did not increase overall detection of high-grade dysplasia (HGD) or cancer and did not eliminate the need for random sampling [22].

#### 4.1.2. Narrow-Band Imaging

Narrow-band imaging (NBI) is a form of virtual (dye-free) chromoendoscopy that filters white light into narrow blue (415 nm) and green (540 nm) bands, preferentially absorbed by hemoglobin, sharpening the contrast of superficial mucosal and vascular patterns without applying dye [23]. These patterns can be classified to predict the underlying histology. The Barrett’s International NBI group (BING) criteria, developed and validated by an expert panel across centers in the United States, Europe, and Japan, predicted dysplasia with 85% accuracy, 80% sensitivity, 88% specificity, and an 88% negative predictive value (NPV); when applied with high confidence, accuracy rose to 92% and specificity to 93%, with substantial interobserver agreement (kappa = 0.68) [24]. In a subsequent international randomized controlled trial, NBI-targeted biopsy matched HD-WLE with the Seattle protocol for intestinal metaplasia detection while requiring significantly fewer biopsies and identifying more patients with dysplasia; areas with a regular NBI pattern harbored no HGD or cancer, suggesting that biopsies may be safely avoided in such areas [25].

#### 4.1.3. Chromoendoscopy

Among dye-based methods, acetic acid chromoendoscopy (AAC) is the most clinically compelling. Dilute acetic acid (1.5–3%) applied to the segment produces a transient acetowhitening reaction through reversible protein denaturation; dysplastic and neoplastic foci, which contain fewer cytoplasmic proteins, lose this whitening early and focally (appearing as red areas against pale mucosa), guiding targeted biopsy. In a meta-analysis of nine studies (1379 patients), AAC detected HGD or early cancer with a pooled sensitivity of 92% (95% CI, 83–97) and specificity of 96% (95% CI, 85–99), with a positive likelihood ratio of 25.0, indicating a substantially raised post-test probability when positive [26]. AAC requires no equipment beyond a standard endoscope, enhancing its applicability in resource-limited settings. Methylene blue and indigo carmine are alternative dyes but generally perform less well than AAC [27].

### 4.2. Optical Biopsy Techniques

#### 4.2.1. Confocal Laser Endomicroscopy

Confocal laser endomicroscopy (CLE) enables real-time, in vivo microscopy of the mucosa at roughly 1000-fold magnification during ongoing endoscopy, following intravenous fluorescein as a contrast agent. Endoscope-integrated (eCLE) and probe-based (pCLE) platforms both resolve crypt architecture, goblet-cell density, and microvasculature that correlate closely with conventional histology. In the first BE series, applying the Mainz criteria, eCLE predicted BE and BE-associated neoplasia with sensitivities of 98.1% and 92.9% and specificities of 94.1% and 98.4%, respectively [28]. A subsequent multicenter randomized controlled trial found that adding eCLE to HD-WLE almost tripled the diagnostic yield for neoplasia while reducing the number of biopsies required [29]. However, adoption remains constrained by cost, a narrow field of view, and a requirement for dedicated operator training, confining CLE largely to tertiary referral centers.

#### 4.2.2. Optical Coherence Tomography and Volumetric Laser Endomicroscopy

Optical coherence tomography (OCT) applies principles analogous to B-mode ultrasound but uses infrared light rather than sound to generate cross-sectional images of tissue architecture to a depth of approximately 2 mm. Volumetric laser endomicroscopy (VLE; Nvision VLE, NinePoint Medical) is a second-generation, commercially available OCT platform that acquires a 6 cm circumferential scan of the entire segment in about 90 s, enabling subsurface assessment and detection of buried glandular mucosa beneath neo-squamous epithelium after ablation—a recognized blind spot of surface-based modalities. In a systematic review and meta-analysis, OCT detected HGD or EAC with a pooled per-lesion sensitivity of 89% and specificity of 91%, whereas VLE achieved 85% and 73%, respectively [30]. The Mayo Clinic VLE diagnostic algorithm, which scores loss of tissue layering and glandular architecture, yielded a sensitivity of 86% and specificity of 88% for dysplasia [31]; VLE-guided laser cautery marking further allows suspicious sites to be tagged for precise, co-registered biopsy [32].

In summary, advanced endoscopic imaging now extends well beyond the Seattle protocol, with each modality contributing complementary strengths. Measured against the ASGE Preservation and Incorporation of Valuable Endoscopic Innovations (PIVI) thresholds for replacing random biopsy (per-lesion sensitivity ≥ 90%, NPV ≥ 98%, and specificity ≥ 80% for HGD/EAC), NBI and AAC are currently the most practice-ready technologies [33], whereas CLE and VLE remain specialized tools used mainly in expert centers for lesion characterization and post-ablation surveillance. The diagnostic performance of the major modalities is summarized in Table 2.

## 5. Biomarker-Based Diagnosis of Barrett Esophagus

Even with high-quality endoscopy and the advanced imaging described above, the following two problems persist that better visualization alone cannot resolve: the diagnosis still turns on a subjective histologic grade, and most patients who develop esophageal adenocarcinoma (EAC) were never known to have Barrett’s esophagus (BE) beforehand. What the field needs, alongside a clearer view of the mucosa, is a way to read the underlying biology—objective, reproducible markers that can confirm the diagnosis of BE and stratify the risk of neoplastic progression. The ideal panel would span diagnostic, predictive, and prognostic roles as follows: establishing disease, estimating the likelihood of malignant transformation, and forecasting therapeutic outcome and survival in EAC [34]. Despite decades of work across genomic, epigenetic, and proteomic platforms, no single marker has yet replaced histologic dysplasia grading as the clinical standard, but the field is moving quickly, and its most promising momentum comes from pairing molecular panels with minimally invasive sampling devices.

### 5.1. Molecular and Genetic Biomarkers

#### 5.1.1. p53 and 17p Loss of Heterozygosity

TP53, the gene encoding the tumor-suppressor protein p53, sits at the center of neoplastic progression in BE. One of the earliest events in the metaplasia–dysplasia–adenocarcinoma sequence is loss of heterozygosity (LOH) at the 17p chromosomal locus that harbors TP53, and loss of p53 carries a roughly 16-fold increase in cancer risk [35]. As a screening test, however, p53 immunohistochemistry (IHC) is a blunt instrument: only about 32% of cases that go on to progress stain positive at baseline [35], so the marker is highly specific but too insensitive to catch progression on its own. Where it earns its place is as a diagnostic adjunct: several guidelines now recommend p53 IHC to help adjudicate biopsies reported as indefinite for dysplasia [36].

#### 5.1.2. DNA Content Abnormalities

Aneuploidy and tetraploidy, measured by flow or image cytometry, are among the best-validated tissue biomarkers for risk stratification and can serve as predictive markers in patients with no or low-grade dysplasia [35]. In a landmark long-term follow-up of the Seattle BE cohort, a panel combining 17p LOH, 9p LOH, aneuploidy, and tetraploidy was the single strongest predictor of progression to EAC; patients with all these abnormalities reached a strikingly high cumulative cancer incidence [37]. Reid and colleagues, in an influential framework for reading the biomarker literature, observed that of more than sixty markers proposed at the time, only a handful had reached the phase 3 and 4 studies that matter to the practicing clinician, with flow-cytometric tetraploidy and aneuploidy furthest along, and that the poor cross-institutional reproducibility of dysplasia grading was itself a major barrier to writing national guidelines [38]. In practice, flow cytometry has largely given way to image cytometry, which works on formalin-fixed, paraffin-embedded tissue and therefore fits far more comfortably into routine pathology workflows.

#### 5.1.3. Biomarker Stability

Part of why aneuploidy has held up so well is its stability. In a systematic study that sampled aneuploidy alongside proliferation markers (Ki67, Mcm2) and cell-cycle markers (cyclin D1, cyclin A) at three levels of the BE segment across two serial endoscopies, aneuploidy was the only marker whose spatial and temporal variability stayed below 10% [39]. Other markers swung by as much as 90%, even in patients who never progressed [39]—a reminder that where and when a biopsy is taken can matter as much as the marker itself. This has real consequences for both trial design and clinical practice as follows: a marker that varies widely across the segment may need to be sampled at several sites or captured by wide-area techniques, before it can be trusted for risk assessment [39]. That problem is precisely what the non-endoscopic devices discussed below were built to solve.

#### 5.1.4. Fluorescence in Situ Hybridization

FISH offers a further way to detect locus-specific DNA abnormalities. A four-probe set has identified dysplasia, high-grade dysplasia, and EAC with a sensitivity of 84–93% and a specificity of 93% [35]. Its cost, technical demands, and need for specialized laboratory infrastructure have nonetheless kept it from wide clinical use.

### 5.2. Epigenetic Markers

Promoter hypermethylation is one of the earliest molecular events in BE carcinogenesis, and its detectability in both tissue and cell-free DNA makes it an appealing biomarker platform. The most studied target is the cell-cycle inhibitor p16 (CDKN2A): hypermethylation and loss of p16 independently raise the risk of progression to high-grade dysplasia [35], but p16 methylation is so common in BE, reported in 34–66% of cases [35], that it is of little use as a stand-alone discriminator. Its value emerges only in combination with other markers.

That realization has pushed the field from single genes toward multi-gene methylation panels. In a multicenter, double-blinded validation study of 195 patients, an eight-gene panel (p16, RUNX3, HPP1, NELL1, TAC1, SST, AKAP12, and CDH13) predicted progression with a sensitivity near 50% [40]. Fifty percent is still too low to screen on, but the panel clearly outperformed any single marker, and the arrival of next-generation sequencing has since opened up epigenome-wide methylation profiling. As Huang and Hardie observed, the range of epigenetic and proteomic changes detectable in Barrett’s tissue is enormous; the bottleneck has never been candidate markers, but the large-scale prospective validation needed to make them clinically actionable [41]—the very gap that panel-based approaches are beginning to fill. If there is one lesson that runs through this whole body of work, it is that panels succeed where individual markers fall short.

Methylation panels carry one more practical advantage: because they can be read from non-endoscopic samples—Cytosponge specimens or cell-free DNA from blood or urine—they slot neatly into the endoscopy-sparing strategies now taking shape in the clinic.

### 5.3. Cytosponge

The most striking recent advance in this area is the Cytosponge, a swallowable cell-collection device that samples the esophagus in a wide-area, non-targeted way, fundamentally overcoming the sampling limitations of protocol biopsies. It is a 3 cm sphere of medical-grade polyester mesh on a string, packed into a gelatin capsule. The patient swallows the capsule while holding the string; after about five minutes the gelatin dissolves, the sphere expands, and it is drawn back up through the esophagus, gathering cells along the entire segment on the way out [42]. The specimen is stained for trefoil factor 3 (TFF3), a protein secreted at the luminal surface of intestinal metaplasia but absent from the neighboring squamous epithelium, which makes it a clean marker for the presence of BE [35]. The device was shown to be safe, acceptable, and diagnostically accurate first in the BEST2 case–control study and then in the larger BEST3 randomized trial [42,43].

It is worth being explicit about what the current evidence does and does not establish. The Cytosponge-TFF3 pathway is designed as a screening and case-finding tool. American College of Gastroenterology (ACG) has suggested the use of swallowable, nonendoscopic capsule sponge device combined with biomarker as an acceptable alternative to endoscopy for BE screening (conditional recommendation with very-low quality of evidence). It is not a substitute for endoscopic surveillance in patients with an established BE diagnosis, where biopsy-based histology remains the reference standard for detecting dysplasia and guiding treatment decisions.

Emerging data support the use of Cytosponge as a triage tool when combined with biomarkers—p53 overexpression and cytologic atypia—both of which are read from the same sample. These markers were developed and validated in a separate multicenter, retrospective cross-sectional cohort of patients already undergoing endoscopic surveillance for established BE—a distinct population from the primary-care screening cohorts of BEST2 and BEST3. Applied to this training and validation data, these markers sorted patients into the following three tiers: high risk (atypia, p53 overexpression, or both on Cytosponge), moderate risk (a clinical risk factor such as age, sex, or segment length), and low risk (Cytosponge-negative with no clinical risk factors) [44]. The separation was clinically meaningful as follows: the risk of high-grade dysplasia or intramucosal cancer in the high-risk group was 52% in the training cohort and 41% in the validation cohort, against just 2% and 1% in the low-risk group [44]. When the same decision tree was tested prospectively in the real-world DELTA cohort, set up partly to relieve endoscopy backlogs during the COVID-19 pandemic, for surveillance-triage population and not primary screening, it classified 39 of 223 patients (17%) as high risk requiring endoscopy; of these, 12 had high-grade dysplasia or intramucosal cancer (positive predictive value [PPV] 31%) and 17 had dysplasia of any grade (PPV 44%) [44]. These counts make the downstream endoscopy burden concrete: of the 39 high-risk patients who underwent endoscopy, 22 did not have dysplasia of any grade. This is an expected, and in a triage context acceptable, trade-off of prioritizing a higher-risk subgroup rather than surveilling the whole population by endoscopy directly, but it means the pathway does not eliminate downstream endoscopy demand—it redistributes and concentrates it.

Implementation data on a much larger scale, within the context of surveillance, have since reinforced the point. Across 10,577 Cytosponge procedures performed in 61 hospitals in England and Scotland, 8.3% of surveillance samples carried dysplastic biomarkers (4.0% for p53 and 7.6% for atypia), rising to 19.7% in ultra-long segments [45]. In other words, a scalable, laboratory-based biomarker panel can steer scarce endoscopy capacity toward the patients most likely to benefit—a genuinely different model for population-level BE surveillance than one built entirely on endoscopy.

Generalizability beyond this specific setting is not yet established. The supporting studies—BEST2, BEST3, the DELTA cohort, and the 61-hospital implementation program—were all conducted within the English and Scottish NHS, but they do not represent a single uniform population: BEST2 and BEST3 recruited patients referred through UK primary care pathways for reflux symptoms, whereas the DELTA cohort instead enrolled patients with established BE undergoing endoscopic surveillance. How the test performs, and how triage thresholds should be calibrated, in other healthcare systems, in populations with different ethnic and genetic backgrounds, or in truly asymptomatic screening rather than symptom-triggered case-finding or surveillance-based triage, remains to be demonstrated. Implementation also depends on infrastructure that does not yet exist everywhere: centralized or standardized cytopathology capacity for TFF3, p53, and atypia scoring, quality-assurance programs across reading sites, clear referral pathways from primary care into confirmatory endoscopy, and, as with any new sampling device, an inadequate-sample rate that adds a small but real repeat-testing burden.

The caveats are real. Cytosponge is less sensitive to short-segment than for long-segment disease, and a real-world positive predictive value around 31% for high-grade dysplasia means a fair number of patients sent for endoscopy will turn out not to have significant neoplasia. While the progress in biomarker discovery, assay development, and minimally invasive sampling has been genuinely impressive [46], the case for adopting this approach at the guideline level will ultimately rest on how well the accumulating trial-based cost-effectiveness evidence holds up as it is tested against real-world uptake and broader populations.

Cost-effectiveness evidence has, in fact, begun to accumulate, though important uncertainties remain. Early microsimulation modeling of a hypothetical cohort estimated an incremental cost-effectiveness ratio (ICER) of roughly $15,700 per quality-adjusted life-year (QALY) for Cytosponge screening followed by treatment of symptomatic patients compared with no screening—more favorable than modeled endoscopic screening. A subsequent economic evaluation built directly on BEST3 trial data, rather than a hypothetical cohort, estimated an ICER of about £5500 (roughly $7500) per QALY, with a greater than 90% probability of cost-effectiveness at a £20,000 (roughly $27,200)/QALY willingness-to-pay threshold [47,48]. That trial-based analysis, however, used a relatively low real-world uptake (24%) and an older trial population than earlier models assumed, either of which could shift the estimate if uptake or the target age range changes in practice; it is also specific to UK NHS costs and care pathways, and comparable prospective economic evaluations from other health systems, or for the biomarker-panel triage pathway specifically rather than the base TFF3 test, are still lacking. Cost-effectiveness at the level of an individual health system therefore remains a genuine, unresolved question rather than a settled one.

In summary, no single biomarker has replaced dysplasia grading, but the shape of a better system is now visible. DNA content abnormalities, aneuploidy in particular, remain the most robust and reproducible tissue markers; p53 IHC has found a defined role in resolving equivocal biopsies; and methylation panels have shown that combinations outperform any marker alone. The most consequential shift, though, is the marriage of these biomarkers to non-endoscopic sampling: the Cytosponge, coupled with TFF3, p53, and atypia, has moved from proof of concept to real-world triage at the scale of tens of thousands of patients. What now stands between these tools and routine guideline adoption is less a matter of biological insight than of prospective validation and health-economic evidence.

## 6. Non-Endoscopic and Minimally Invasive Approaches

Conventional upper endoscopy (esophagogastroduodenoscopy, EGD) with four-quadrant biopsy remains the reference standard for the diagnosis of Barrett esophagus (BE), yet its reliance on sedation, dedicated endoscopy facilities, and appreciable cost has kept screening uptake low. In a screen-eligible primary care population, only 39% of patients had undergone EGD and only 9.2% were referred specifically for BE screening [49]. This shortfall has motivated the development of non-endoscopic and minimally invasive alternatives that seek to preserve diagnostic accuracy while lowering the procedural and economic burden of screening. This section considers the following two broad strategies: (A) capsule-based esophageal visualization and (B) biomarker-based approaches using exhaled breath analysis and blood-based liquid biopsy.

### 6.1. Esophageal Capsule Endoscopy

Esophageal capsule endoscopy (ECE) uses a swallowable, wireless capsule bearing miniature cameras at both poles that photograph the mucosa simultaneously as the device is carried through the esophagus by peristalsis. First-generation PillCam ESO capsules transmitted images at 4 frames per second, a rate soon increased to 14 frames per second to keep pace with the rapidly traversed esophagus [50]; the second-generation PillCam ESO 2 further raised the capture rate to 15 frames per second while widening the field of view [51]. Because the examination requires neither sedation nor air insufflation, it is well-tolerated and can be completed within a few minutes.

#### 6.1.1. Diagnostic Performance

In a meta-analysis of nine blinded studies enrolling 618 subjects, ECE detected BE with a pooled sensitivity of 77% and specificity of 86% [52]. Sensitivity and specificity were 78% and 90% when endoscopy alone served as the reference standard, but specificity fell to 73% when histologic confirmation of intestinal metaplasia was required [52]. Substantial heterogeneity in specificity across studies (I^2^ = 74%) reflected differences in capsule generation, ingestion protocol, and reader experience [52]. The defining limitation of ECE, common to every study, is that it cannot obtain tissue: intestinal metaplasia can be suspected but never confirmed, so any positive finding still mandates a confirmatory EGD with biopsy. This two-step pathway erodes much of the practical advantage the technique is meant to provide.

#### 6.1.2. Cost Effectiveness

Two Markov analyses reached broadly concordant conclusions. Modeling lifetime costs and life expectancy for 50-year-old men with chronic gastroesophageal reflux disease (GERD), Gerson and Lin found that an initial EGD-with-biopsy strategy cost $1988 and yielded 18.54 life-years, against $2392 and 18.36 life-years for a capsule-endoscopy-first strategy; EGD cost $4530 per life-year gained relative to no screening [53]. In a societal cost-utility analysis, Rubenstein and colleagues estimated EGD at $11,254 per quality-adjusted life-year (QALY) gained while averting 60% of esophageal adenocarcinoma (EAC) deaths, whereas ECE prevented only 53% of cancers, cost more overall, and returned nine fewer quality-adjusted days [54]. In both models, EGD dominated ECE, principally because inconclusive capsule studies generate downstream confirmatory endoscopies [53,54].

### 6.2. Breath Testing and Liquid Biopsy

#### 6.2.1. Exhaled Volatile Organic Compound Profiling

Breath-based testing rests on the premise that the altered metabolism of Barrett mucosa yields a distinctive profile of exhaled volatile organic compounds (VOCs). The following two analytic approaches predominate: gas chromatography-mass spectrometry (GC-MS), which examines individual compounds, and the electronic nose (e-nose), which uses sensor arrays and machine-learning classifiers to recognize the aggregate VOC signature. In a case–control study of 66 patients with BE and 56 controls, an e-nose distinguished the groups with 82% sensitivity, 80% specificity, and an area under the receiver-operating-characteristic curve (AUROC) of 0.79 [55]. Peters and colleagues subsequently developed and cross-validated an e-nose prediction model in 402 patients, achieving 91% sensitivity and 74% specificity; performance was independent of proton-pump-inhibitor use, hiatal hernia, and reflux severity, supporting its potential as a first-line triage test [56]. Despite these encouraging results, breath analysis is not yet ready for clinical screening, as standardized protocols and external validation are still lacking.

#### 6.2.2. Blood-Based Liquid Biopsy

Liquid biopsy detects tumor- or metaplasia-derived nucleic acids, such as microRNA (miRNA) and methylated cell-free DNA, circulating in peripheral blood. Requiring neither a swallowed device nor sedation, it is uniquely suited to large-scale screening. The EMERALD (Esophageal MicroRNAs of BaRRett, Adenocarcinoma, and Dysplasia) study is the largest blood-based liquid-biopsy investigation in BE to date, analyzing 792 patient samples from four countries [57]. Integrating tissue-based miRNA sequencing with iterative validation, the investigators identified six circulating miRNAs (miR-106b, miR-146a, miR-15a, miR-18a, miR-21, and miR-93) consistently overexpressed across BE, dysplasia, and EAC relative to non-disease controls [57]. A machine-learning classifier (a stacked XGBoost and AdaBoost model) combining these markers achieved an AUROC of 97.6% in the training cohort and 91.9% in an independent external test cohort; for the clinically decisive task of separating BE from symptomatic GERD controls, it reached an AUROC of 94.8%, with 92.8% sensitivity and 85.1% specificity [57]. A companion microsimulation modeling five screening programs in a 45-year-old GERD population over 30 years suggested that EMERALD testing at 45% participation would detect EAC substantially earlier than endoscopic screening limited to 10% participation, though prospective validation is still required [57].

#### 6.2.3. Relationship to Cytosponge-TFF3

Among cellular, rather than truly blood-based, sampling methods, the Cytosponge-TFF3 platform remains the most clinically mature non-endoscopic option and provides a useful benchmark for the modalities above. Because it is examined in detail in the preceding section on biomarker-based diagnosis, it is not re-described here and is retained in Table 3 only for comparison [42,43].

Hence, the non-endoscopic and minimally invasive tests differ markedly in clinical readiness (Table 3). ECE offers sedation-free direct visualization but, under base-case economic modeling, costs more than EGD because positive or inconclusive studies still require confirmatory endoscopy [52,53,54]. The Cytosponge-TFF3 test is the best-validated non-endoscopic approach, supported by a large multicenter randomized trial (detailed in the biomarker section) that markedly increased BE detection over usual care with high patient acceptability [42,43]. Exhaled-VOC analysis by e-nose is sensitive but not yet sufficiently specific (74–80%) to stand alone, so a substantial fraction of positive results would still proceed to confirmatory endoscopy [55,56]. Blood-based liquid biopsy, exemplified by EMERALD, is arguably the most transformative long-term prospect: a simple venous sample, an AUROC of 94.8% for discriminating BE from GERD, and the adherence advantages of a blood test give it the potential to reshape screening once prospective validation is complete [57]. The most rational future pathway is likely sequential risk stratification, in which these complementary non-invasive tests triage patients toward selective endoscopy, maximizing BE detection while minimizing unnecessary procedures.

## 7. Artificial Intelligence in the Diagnosis of Barrett Esophagus

The diagnostic difficulties that recur throughout this review—the operator dependence of endoscopic lesion recognition, the sampling error inherent to biopsy protocols, and the interobserver variability of dysplasia grading—are precisely the problems that artificial intelligence (AI) is positioned to address. Deep learning, and in particular the convolutional neural network (CNN), can learn discriminative mucosal features directly from large image sets and apply them reproducibly, offering a route to more consistent recognition of early neoplasia. Over the past several years AI has advanced from retrospective image classification toward real-time decision support during live endoscopy and, increasingly, toward the interpretation of non-endoscopic samples [58]. This section compares AI-guided and conventional endoscopy, describes the integration of AI into minimally invasive screening, and weighs the benefits and limitations that currently frame AI as an adjunct to, rather than a replacement for, expert practice.

### 7.1. Real-Time Dysplasia Detection: AI-Guided Versus Conventional Endoscopy

Real-time detection of dysplasia in Barrett’s esophagus has traditionally depended on white-light endoscopy supplemented by image-enhanced modalities, in which lesion recognition is strongly operator-dependent. In routine practice the sensitivity of conventional endoscopy for early neoplasia is variable and shaped by endoscopist experience and lesion morphology [58]. Even with systematic four-quadrant sampling under the Seattle protocol, dysplastic foci are frequently focal, flat, and visually subtle, so sampling error remains an important cause of missed lesions during surveillance [58,59].

AI-guided endoscopy addresses these limitations by applying CNNs to live video frames, flagging suspicious regions in real time and functioning as a tireless “second observer” that directs targeted biopsy. In the first prospective demonstration during live procedures, a computer-aided detection system identified neoplasia with 90% accuracy, 91% sensitivity, and 89% specificity on per-level analysis, correctly classifying nine of ten neoplastic patients while generating few false-positive predictions [60]. Subsequent real-time systems have reproduced this performance, with reported sensitivities of roughly 85–96% and specificities of 84–94% and areas under the curve frequently exceeding 0.90 [60,61,62,63].

Pooled analyses confirm these findings at the level of the literature as a whole. Systematic reviews report an overall AI sensitivity near 89–90% and specificity around 84–86% for the detection of Barrett’s neoplasia, consistently exceeding unassisted assessment [64,65,66]. The benefit is greatest where it is most needed: AI narrows the performance gap between non-expert and expert endoscopists and reduces interobserver variability, effectively distributing specialist-level pattern recognition to lower-volume settings [65,66].

Beyond the CNN-based, frame-by-frame classification systems described above, a separate line of work has begun to combine object-detection architectures with hyperspectral imaging to add spectral, not just spatial, information to lesion detection. Weng et al. transformed conventional endoscopic images into hyperspectral-like data using a Spectrum-Aided Visual Enhancer (SAVE) and benchmarked six YOLO-family object-detection models (YOLOv3 through YOLOv8) for identifying esophageal dysplasia and squamous cell carcinoma; the hyperspectral-derived data improved detection over conventional white-light images across most models, with YOLOv8 accuracy for dysplasia rising from 80.9% with white-light imaging to 82.1% with the SAVE-derived hyperspectral data [67]. Yang et al. applied a similar YOLOv5-based object-detection pipeline to a larger dataset of white-light and narrow-band endoscopic images for early esophageal cancer detection, reinforcing that pairing object-detection architectures with hyperspectral or spectrally enhanced imaging is a reproducible way to improve overall detection performance [68]. Both studies illustrate a direction that extends beyond the traditional image-classification framing of most Barrett-specific AI work discussed above—treating lesion identification as an object-detection problem in spectrally enriched image space—though this evidence base, like the SAVE/hyperspectral literature more broadly, is derived largely from single-institution Taiwanese cohorts studying esophageal cancer and dysplasia in general (predominantly squamous-cell disease) rather than Barrett-associated dysplasia or adenocarcinoma specifically; thus, its generalizability to BE surveillance in Western cohorts, where adenocarcinoma predominates, remains to be established.

When judging how close AI-guided detection is to routine use, it should be noted that there are two evidentiary tracks—a handful of prospective, real-time evaluations versus a much larger body of retrospective, image- or frame-level studies. The following three studies have tested a system prospectively during live endoscopy: de Groof et al., Ebigbo et al., and Fockens et al.: the first in a single-center pilot of 20 patients analyzed at the per-level (three images/level), the second comparing the AI system with a single expert endoscopist in 14 patients (36 neoplastic and 26 non-neoplastic image regions) at one German center, analyzed at the per image region, where the system’s sensitivity and specificity were 83.7% and 100.0% [60,61], and a 2024 pilot testing a CADe system live during endoscopy in 30 patients (15 with a visible lesion, 15 without), which reported a per-patient sensitivity of 100% but a per-patient specificity of only 53% (per-level sensitivity and specificity of 100% and 73%) [69]. That third study is discussed further below in the context of false-positive burden and real-world readiness. The remaining evidence—the narrative and systematic reviews [62,63] and the pooled meta-analyses [64,65,66]—synthesizes the substantially larger literature, but one still dominated by retrospective analysis of curated, expert-annotated still images or video clips rather than prospective, real-time, patient-level testing; the pooled sensitivity and specificity figures cited above are therefore best read as an estimate of image-classification performance under close-to-ideal conditions, not as a validated real-world, per-patient detection rate.

In summary, heterogeneity in training datasets, differences in image quality between endoscopy systems, and the gap between curated study images and unselected real-world video all constrain generalizability [64]. Reported performance therefore represents an upper bound that may not transfer directly to community practice and prospective external validation across diverse platforms remains limited.

### 7.2. AI Integration with Non-Endoscopic Sampling

AI is not confined to the endoscopy suite; it is increasingly paired with minimally invasive sampling to extend screening beyond specialist centers. The Cytosponge, a swallowed capsule-sponge that retrieves esophageal cells for trefoil factor 3 (TFF3) immunostaining, still depends on pathologist review, which limits throughput. Machine-learning quantification of TFF3 from these samples has been shown to predict clinically relevant Barrett’s esophagus and to prioritize slides for expert attention [70]. Extending this approach, a weakly supervised deep-learning model trained on routinely stained hematoxylin-eosin whole-slide images identified Barrett’s esophagus with an area under the curve of approximately 0.93 across two clinical-trial cohorts, without the localized expert annotation such models usually require [71]. By automating the initial read and triaging higher-risk cases, these tools address the pathology bottleneck that would otherwise constrain population-scale, non-endoscopic screening.

### 7.3. Benefits, Limitations, and Challenges

Across both endoscopic and non-endoscopic applications, the benefits of AI are consistent: higher sensitivity for early neoplasia, fewer missed lesions, more standardized interpretation, and reduced interobserver variability, together with the potential to democratize expertise by bringing specialist-level performance to non-expert operators [72,73]. These advantages are counterbalanced by real limitations. Model performance depends heavily on the quality and representativeness of the training data; external validation is still sparse; and generalizability across endoscopy platforms, patient populations, and community settings is unproven [72]. Practical barriers to adoption include cost, workflow integration, and regulatory approval [73].

External validation deserves particular emphasis. Most of the sensitivity and specificity figures summarized above were generated by testing a model on data held out from the same institution, or occasionally the same country, that produced its training set; independent validation on data from centers, endoscope manufacturers, and patient populations the model has never seen remains the exception rather than the rule. A multi-center UK trial now prospectively evaluating a CAD system for Barrett’s neoplasia across four NHS trusts is a step toward the kind of real-life, multi-site validation this field needs, but results are not yet available, and comparable prospective external-validation studies remain scarce elsewhere [74].

The false-positive burden also deserves explicit attention, because a specificity figure that looks reassuring in isolation translates differently once actual disease prevalence is considered. Non-dysplastic tissue accounts for the large majority of surveillance endoscopies, with dysplasia present in only a minority of examinations; at a pooled AI specificity of roughly 84–86%, a meaningful share of AI-flagged regions in a typical surveillance procedure would still be false positives, each prompting an additional targeted biopsy that would not otherwise have been taken. Fockens et al.’s prospective pilot makes this concrete: despite a per-patient sensitivity of 100%, per-patient specificity was only 53%—in 13 of the 15 patients without a visible lesion, the CADe system nonetheless flagged a suspicious area during the initial pullback, and in 7 of those 13 the false-positive finding persisted on closer, level-based video review [69]. A real-time system deployed on unselected patients may therefore generate a materially higher false-positive rate than the pooled, retrospective, image-level literature suggests, with direct consequences for unnecessary biopsies, procedure time, and endoscopist trust in the system—all central to real-world readiness.

The evidence on non-expert performance is consistent but thin. In the one real-time study that directly compared AI with a single experienced endoscopist, the two disagreed on only one of the examined regions, and the authors framed the result as evidence that AI-based detection could be of particular value for less experienced endoscopists, though the study was neither designed nor powered to quantify that benefit directly [61]. Workflow integration is a related, underexplored barrier: even a well-performing model must be connected to a specific endoscopy tower’s video output and be usable by staff without machine-learning expertise—a setup that might be straightforward in a research-intensive academic center but is a genuine barrier in a busy community endoscopy unit.

Regulatory status is beginning to catch up with the research literature but should not be mistaken for equivalence to it. NEC’s WISE VISION Endoscopy received CE marking for Barrett’s neoplasia detection in 2021—a European authorization, and Olympus commercially launched its OLYSENSE CAD/AI platform in both the US and Europe in 2025, but the regional status differs by application within that platform: CADU, the Barrett’s esophagus dysplasia-detection tool, received CE marking in Europe, while only CADDIE, the platform’s colorectal-polyp-detection tool, received FDA 510(k) clearance in the US [75,76], indicating that AI-guided Barrett’s detection systems have begun to clear a regulatory bar for market authorization in at least one jurisdiction. However, these are two distinct regulatory pathways, not a single “clearance” standard, and neither is a guarantee of the clinical-outcome evidence discussed above. FDA 510(k) clearance, the pathway used for CADDIE, requires the manufacturer to demonstrate substantial equivalence to a legally marketed predicate device—a comparative safety-and-performance standard, not an independent efficacy review. CE marking, the pathway used for both WISE VISION and CADU, is a separate EU conformity-assessment process under the Medical Device Regulation that certifies a device meets EU safety and performance requirements; it is not predicate-based and has no FDA 510(k) equivalent. Under either pathway, regulatory authorization falls short of proof of the large-scale, prospective, multi-center, patient-outcome benefit that would be needed to justify routine, guideline-endorsed use. So, the arrival of commercially available systems narrows, but does not close, the evidence gap described above.

Looking ahead, the principal challenges are large-scale prospective validation, standardization of algorithms and reporting, and resolution of the ethical and data-privacy concerns surrounding the large image datasets these systems require [72,73]. For these reasons, AI in Barrett’s esophagus is best understood at present as an adjunct that augments the endoscopist and pathologist rather than a replacement for either.

## 8. Clinical Function, Guideline Status, and the Patient Journey: A Synthesis

Having surveyed each technology in the sections above, it is useful to step back and make explicit a distinction that has been implicit throughout: these technologies serve genuinely different clinical purposes and grouping them by imaging modality or biomarker class can obscure how differently each one functions within a patient’s actual care pathway. The following five clinical functions recur across this review: (1) screening/case-finding—applying an inexpensive, well-tolerated test to an at-risk population that has not yet undergone endoscopy; (2) confirmation/diagnosis—establishing the histologic presence of BE once a patient reaches endoscopy; (3) dysplasia/neoplasia detection and characterization—identifying and grading focal lesions within an already-established BE segment; (4) progression-risk stratification—estimating the likelihood that non-dysplastic or indefinite disease will progress; and (5) post-ablation surveillance—detecting recurrent or buried disease after endoscopic eradication therapy. These functions carry different target populations and different consequences for a false result and therefore different acceptable performance thresholds as follows: a screening test can tolerate a lower specificity than a test used to decide whether to ablate a segment, because a false positive at screening merely triggers confirmatory endoscopy, whereas a false positive at the ablation decision exposes a patient to an invasive therapy with its own risks.

Mapped onto this framework, the technologies reviewed above can be categorized into the following three evidence tiers: (1) established guideline-supported standards, (2) guideline recognized adjuncts and (3) investigational. Established guideline-supported standards—white-light endoscopy with Seattle-protocol biopsy, high-definition endoscopy, and, as adjuncts meeting the ASGE PIVI thresholds, narrow-band imaging and acetic acid chromoendoscopy—anchor confirmation/diagnosis and dysplasia/neoplasia detection [2,15,33]. These image-enhanced techniques are guideline-recognized adjuncts intended to be used together with, not in place of, a structured (Seattle-protocol) biopsy sampling regimen [10]. p53 immunohistochemistry occupies a similar established adjunct role to routine clinical diagnosis, per the British Society of Gastroenterology guidelines [36], specifically for diagnostic adjudication of indefinite-for-dysplasia histology; however, current ACG and AGA guidance, by contrast, makes no recommendation for or against its routine use as a marker of progression risk [2,10]. For screening/case-finding specifically, Cytosponge-TFF3 belongs in the established/guideline-supported tier rather than the adjunct tier based on the 2022 ACG guideline discussed above in the Section 5.3. This recognition is specific to the screening/case-finding function; it does not establish Cytosponge-TFF3 as a replacement for endoscopic surveillance in patients with already-diagnosed BE [2,42,43], and it does not extend to the same biomarker panel’s separate use for progression-risk triage in patients with already-established BE—a distinct application for which no such guideline recommendation exists and which therefore remains investigational, regardless of its supporting real-world implementation data. The remaining technologies are, by the evidence assembled in this review, investigational rather than guideline-endorsed for the functions in which they are discussed. Confocal laser endomicroscopy and OCT/VLE remain confined to expert centers for dysplasia characterization [28,29,30,31,32], while DNA-content/ploidy analysis and multi-gene methylation panels are the best-validated research markers for progression-risk stratification [37,38,40], but none of them have been incorporated into routine guideline pathways, chiefly because prospective validation against hard clinical endpoints (progression to HGD/EAC) remains limited. Esophageal capsule endoscopy, exhaled-VOC breath testing, and circulating miRNA liquid biopsy (EMERALD) are all investigational screening tools: capsule endoscopy is cost-ineffective relative to EGD in decision-analytic modeling and has been largely superseded [52,53,54]; breath testing lacks a standardized protocol and adequately powered external validation [55,56]; and EMERALD, despite a promising AUROC, awaits the prospective validation, real-world implementation experience, and health-economic evidence that any screening role would require [57]. AI-guided real-time detection is similarly promising but investigational: the point estimates in Table 4 trace substantially to a single-center pilot study of 20 patients, and even the pooled ranges from systematic reviews reflect heterogeneous, largely retrospective, expert-center data with limited prospective external validation across endoscopy platforms and community settings [60,61,62,63,64,65,66]. AI-assisted reading of Cytosponge cytology and Barrett’s histology is at an earlier stage still and remains investigational for its screening/confirmation-support role [70,71].

**Figure 1:**
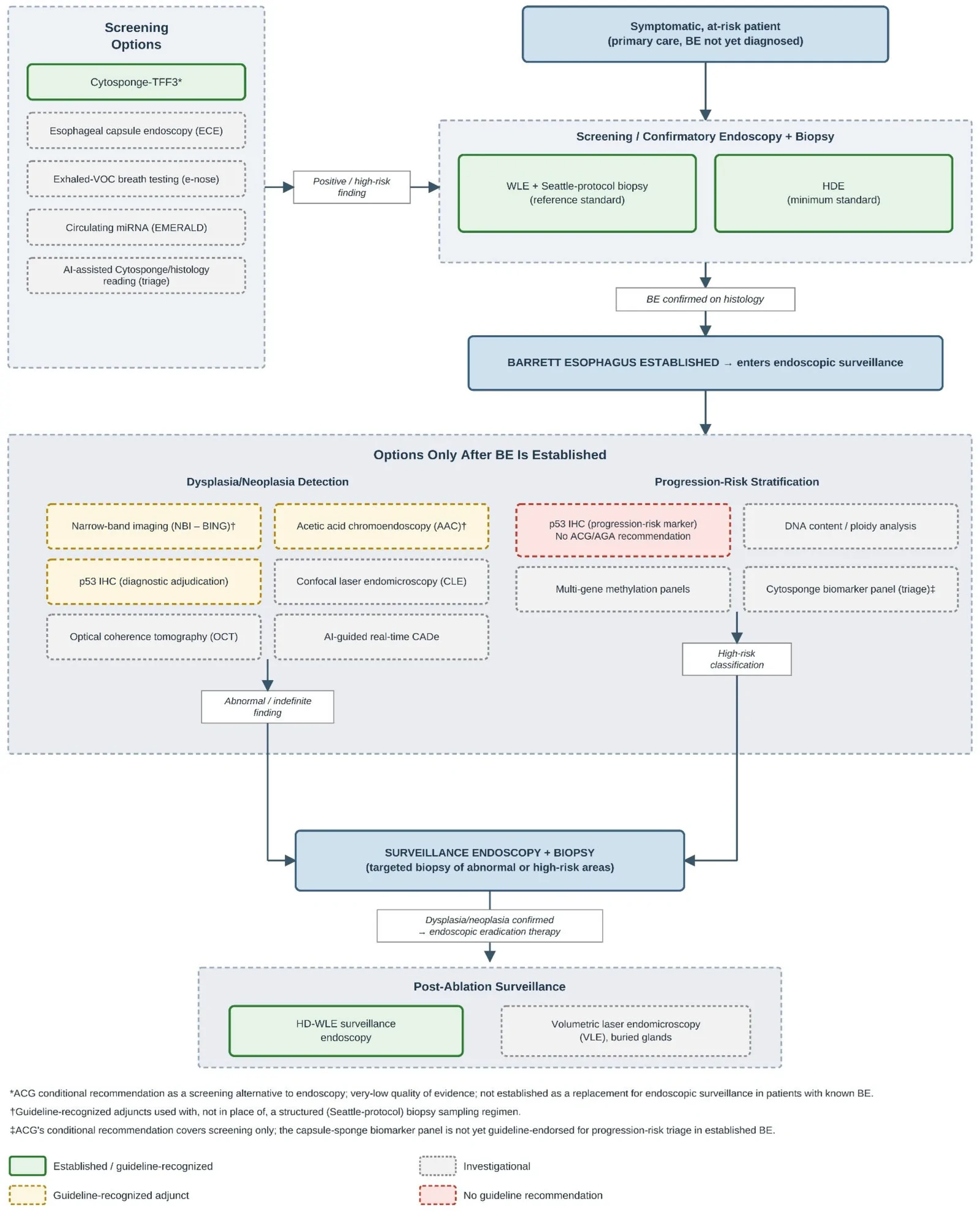
Clinical decision pathway for Barrett esophagus (BE) screening, diagnostic and surveillance technologies. Technologies are grouped by the point in the care pathway at which they are applied (screening, confirmation, dysplasia/neoplasia detection, progression-risk stratification, or post-ablation surveillance) and bordered according to evidence tier: solid border, established/guideline-recognized; dashed border, guideline-recognized adjunct; dotted border, investigational; dashed red border, no guideline recommendation. Several technologies serve more than one function and are cross-referenced accordingly. Abbreviations: AI, artificial intelligence; CADe, computer-aided detection; ECE, esophageal capsule endoscopy; HD-WLE, high-definition white-light endoscopy; miRNA, microRNA; VOC, volatile organic compound; IHC, immunohistochemistry; HDE, high-definition endoscopy.

Table 5 and Figure 1 consolidate this evidence base by clinical function, evidence tier, and position in the care pathway, drawing on the references cited throughout the sections above. Figure 1 renders this explicitly as a clinical decision pathway, showing which positive or high-risk findings lead to confirmatory endoscopy and biopsy, and which technologies apply only after BE has been established. The intent is not to rank technologies against one another—as the preceding sections make clear, their study designs, populations, and units of analysis differ too much for that—but to make explicit which technologies a clinician can rely on today, which are reasonable adjuncts in the right setting, and which, however promising, still require the prospective validation, real-world implementation data, or health-economic evidence that separates an interesting result from a guideline-ready technology. Both the table and the figure are original syntheses constructed by the authors from the evidence and guideline statements discussed throughout this review; neither reproduces a pathway published by any guideline or any other society, and neither should be read as an official or society-endorsed clinical algorithm.

## 9. Future Directions and Emerging Diagnostic Paradigms

The trajectory of Barrett’s esophagus diagnosis is moving away from a single, universally applied endoscopic examination and toward a risk-stratified pathway that reserves endoscopy for those most likely to benefit. Upper endoscopy with biopsy remains the reference standard, but its cost and limited reach leave most patients who develop esophageal adenocarcinoma undiagnosed beforehand [2]. The non-endoscopic and minimally invasive tools discussed in the preceding sections—capsule-sponge cell collection with TFF3 and methylation biomarkers, circulating microRNA and methylated cell-free DNA assays, and exhaled volatile organic compound analysis—are converging on the following common goal: an inexpensive, well-tolerated front-line test that identifies who should proceed to endoscopy [43,56,77]. Deployed at scale, such triage could raise participation, lower cost, and shift detection earlier in the disease course [2,43,56,77].

Artificial intelligence is likely to serve as the connective tissue of this pathway rather than as a standalone modality. Beyond real-time dysplasia detection and lesion characterization at endoscopy [60,61,72], machine-learning models are being developed to read Cytosponge cytology, interpret molecular biomarker panels, and combine them with clinical risk factors to generate individualized risk estimates [70,72]. Integrating heterogeneous inputs—endoscopic imaging, histopathology, molecular markers, and demographics—is a task well suited to AI and could allow surveillance resources to be allocated according to predicted rather than average risk [60,61,70,72].

Underpinning both trends is a broader shift toward precision medicine in Barrett’s esophagus, in which advances in molecular diagnostics and multi-omics profiling sharpen the estimate of who will progress to dysplasia or adenocarcinoma [38,78]. A mature diagnostic paradigm might combine clinical risk factors, advanced imaging, biomarker panels, and AI-assisted analysis into a single stratified framework that intensifies surveillance for high-risk patients while sparing low-risk individuals from unnecessary endoscopy [38,78]. Realizing this vision, however, will require large prospective cohorts, rigorous external validation, and formal cost-effectiveness analysis before these technologies can be recommended for routine clinical practice [2,78].

## 10. Conclusions

Barrett’s esophagus remains the principal identifiable precursor of esophageal adenocarcinoma, a cancer whose incidence has risen sharply and whose prognosis, once symptomatic, is poor [3,6]. The central premise of surveillance—that intercepting the metaplasia–dysplasia–carcinoma sequence can early prevent cancer death—depends entirely on the accuracy of diagnosis, and it is here that conventional practice has shown its limits. Upper endoscopy with Seattle-protocol biopsy remains the reference standard, yet sampling error, interobserver variability in dysplasia grading, and a substantial burden of missed neoplasia mean that a single white-light examination is an imperfect gatekeeper [2,15,19,20]. The advances surveyed in this review are best understood as a coordinated response to these three weaknesses.

Each successive technology addresses a specific gap. Advanced endoscopic imaging—high-definition systems, narrow-band imaging, and acetic acid chromoendoscopy—sharpens the detection of subtle, flat lesions, while optical-biopsy tools such as confocal laser and volumetric laser endomicroscopy bring microscopic assessment into real time [21,24,26,28,30]. Image-enhanced endoscopy improves targeted detection while remaining complementary to structured biopsy sampling. Molecular, genetic, and epigenetic biomarkers add an objective, reproducible read on the underlying biology, with DNA-content abnormalities and multi-gene methylation panels beginning to stratify the risk of progression that histology alone cannot [37,38,40]. Perhaps the most consequential shift is the pairing of these biomarkers with non-endoscopic sampling: the Cytosponge coupled with TFF3, p53, and atypia has moved from concept to real-world triage at the scale of tens of thousands of patients [42,43,44], and blood-based liquid biopsy, exemplified by the EMERALD microRNA panel, points toward screening from a simple venous sample [57]. Cutting across all of these, artificial intelligence has emerged less as a competing modality than as a unifying layer that standardizes interpretation, narrows the gap between expert and non-expert readers, and integrates heterogeneous inputs into individualized risk estimates [60,64,73].

Taken together, these developments describe a clear trajectory as follows: away from a one-size-fits-all endoscopic examination and toward a risk-stratified, multimodal, and increasingly minimally invasive paradigm in which inexpensive front-line tests identify who truly needs endoscopy and precision tools concentrate scarce resources on those most likely to progress [2,78]. That vision is not yet ready for wholesale adoption. Most emerging modalities still require large prospective cohorts, external validation across diverse populations and platforms, and rigorous cost-effectiveness analysis before they can enter routine guidelines [46,57,73]. Nonetheless, the direction of travel is unmistakable. As these complementary technologies mature and converge, the diagnosis of Barrett’s esophagus is poised to become earlier, more accurate, and more equitable—offering a realistic prospect of reducing the incidence and mortality of esophageal adenocarcinoma through detection of disease at its most treatable stage.

## Figures and Tables

**Table 1:** Annual risk of progression to esophageal adenocarcinoma.

Histology	Risk of Cancer (per Year)
Non-dysplastic Barrett esophagus	0.2–0.5%
Barrett esophagus with low-grade dysplasia	0.7%
Barrett esophagus with high-grade dysplasia	7%

**Table 2:** Diagnostic performance of advanced endoscopic imaging modalities in Barrett esophagus.

Modality	Study Design (N)	Target	Reference Standard	Analysis Level	Odds Ratio (OR)	Sensitivity (%)	Specificity (%)	Ref.
High-definition endoscopy (HDE)	Retrospective single-center cohort (435 patients; 521 procedures)	Any dysplasia (targeted)	Histopathologic assessment of the biopsies (comparator: SD-WLE)	Per-patient	3.27 (95% CI: 1.27–8.40) vs. SD-WLE	-	-	[22]
Narrow-band imaging (NBI)—BING criteria	Multicenter expert-panel development/validation (97 patients; 170 images)	Dysplasia/HGD/EAC	Histopathologic assessment of the biopsies	Per-image	-	80 (95% CI: 75.6–85.1)	88 (95% CI: 85.4–91.4)	[24]
Acetic acid chromoendoscopy (AAC)	Meta-analysis (9 studies; 1379 patients)	HGD/early cancer	Histopathologic assessment of the biopsies	Pooled (mixed)	-	92 (95% CI: 83–97)	96 (95% CI: 85–99)	[26]
Confocal laser endomicroscopy (eCLE)—Mainz	Single-center prospective series (63 patients; 11,520 confocal images)	BE/BE-assoc. neoplasia	Histopathologic assessment of the biopsies	Per examined area	-	98.1/92.9	94.1/98.4	[28]
Optical coherence tomography (OCT)	Systematic review/meta-analysis (14 studies; 721 patients; 1565 lesions)	HGD/EAC	Histopathologic assessment of the biopsies	Per-lesion	-	89 (95% CI: 60–98)	91 (95% CI: 47–99)	[30]
Volumetric laser endomicroscopy (VLE)	Systematic review/meta-analysis (14 studies; 721 patients; 1565 lesions)	HGD/EAC	Histopathologic assessment of the biopsies	Per-lesion	-	85 (95% CI: 75–91)	73 (95% CI: 52–87)	[30]
VLE–Mayo (Leggett) algorithm	Single-center comparative diagnostic-performance study (27 patients)	Dysplasia	Histopathologic assessment of the biopsies	Per-lesion	-	86 (95% CI: 69–96)	88 (95% CI: 60–99)	[31]

Paired values (e.g., 98.1/92.9) correspond to the two listed targets, respectively. BE, Barrett esophagus; BING, Barrett’s International NBI Group; CI, confidence interval; EAC, esophageal adenocarcinoma; eCLE, endoscope-based confocal laser endomicroscopy; HGD, high-grade dysplasia; OR, odds ratio; SD-WLE, standard-definition white-light endoscopy; -, not reported. Rows differ substantially in study design, target population, and unit of analysis; these figures should not be ranked against one another as though drawn from a common study design or population.

**Table 3:** Comparative diagnostic performance of non-endoscopic modalities versus standard EGD for Barrett esophagus, categorized by different studies.

Modality	Study Design (N)	Sensitivity (%)	Specificity (%)	Cost vs. EGD
Standard EGD (reference)	Reference standard	N/A *	N/A *	Reference standard
Esophageal capsule endoscopy (PillCam ESO)	Meta-analysis (9 blinded studies; 618 subjects) [52]	77 (pooled) [52]	86 (pooled) [52]	Higher than EGD in Markov models ($2392 vs. $1988/patient) [53,54]
Cytosponge-TFF3	Multicenter case–control (11 UK hospitals; n = 1110: 647 BE, 463 controls) [42]	79.9 (95% CI: 76.4–83.0) overall; 87.2 (95% CI: 83.0–90.6) for ≥3 cm circumferential BE [42]	92.4 (95% CI: 89.5–94.7) [42]	Modeled vs. usual care, not vs. EGD (BEST3-based cost-utility) †
Electronic nose (Chan et al.)	Single-center cross-sectional (n = 122: 66 BE, 56 controls) [55]	82 [55]	80 [55]	Investigational; no formal cost analysis
Electronic nose (Peters et al.)	Single-center proof-of-principle/cross-validation cohort (n = 402) [56]	91 [56]	74 [56]	Investigational; no formal cost analysis
Circulating miRNA (EMERALD)	Multicenter biomarker cohort, discovery + validation (792 samples; 4 countries) [57]	92.8 (BE vs. GERD) [57]	85.1 [57]	Favorable Markov model at 45% participation; validation pending [57]

* EGD/biopsy serves as the accepted reference standard against which every other row in this table is judged; sensitivity and specificity are therefore not applicable to this row and are not reported. Abbreviations: EGD, esophagogastroduodenoscopy; BE, Barrett esophagus; TFF3, trefoil factor 3; GERD, gastroesophageal reflux disease; miRNA, microRNA; RCT, randomized controlled trial; N/A, not applicable. Diagnostic-accuracy metrics reference histology obtained at EGD/biopsy and are reported at the per-patient level throughout; study designs and populations nonetheless differ substantially across rows (case–control vs. cross-validation cohort vs. pooled meta-analysis). The cost figures for esophageal capsule endoscopy are outputs of decision-analytic (Markov) economic models rather than empirical diagnostic-accuracy data and are not directly comparable to the sensitivity/specificity estimates in this table. Given these differences, modalities should not be ranked against one another based on this table alone. † For Cytosponge-TFF3, a BEST3 trial-based cost-utility analysis versus usual care has been reported, with an ICER of approximately £5500/QALY [47,48]; this is a UK-specific estimate and is not a direct economic comparison with EGD, and generalizability to other health systems, as well as the economics of the separate biomarker-based surveillance-triage pathway, remains uncertain. The approximately tenfold increase in Barrett esophagus detection versus usual care is a separate, clinical-yield outcome of the BEST3 randomized trial [43], discussed in the accompanying text. Diagnostic accuracy, clinical yield, and modeled economic outcomes are therefore distinct categories of evidence in this table and should not be conflated.

**Table 4:** Study-level evidence for AI-assisted detection of early neoplasia in Barrett esophagus.

Study	Design and Setting	Sample Size	Unit of Analysis	Reference Standard	Results
de Groof et al., 2020 [60]	Single-center, live, in vivo pilot during endoscopic procedures (the Netherlands)	20 patients (10 non-dysplastic BE, 10 neoplasia); 48 levels analyzed	Per-level (3 images/level)	Expert assessment and histopathology	Accuracy 90%; sensitivity 91%; specificity 89%
Ebigbo et al., 2020 [61]	Single-center, real-time AI vs. one expert endoscopist during live endoscopy (Germany)	14 patients; 36 neoplastic and 26 non-neoplastic image regions	Per-image-region	Histopathology	Accuracy 89.9% Sensitivity 83.7%; specificity 100.0%
Fockens et al., 2024 [69]	Single-center, live, real-time CADe-assisted pilot during endoscopic procedures (the Netherlands)	30 patients (15 with a visible lesion, 15 without)	Per-patient and also per-level	Expert assessment and histopathology	Per-patient sensitivity 100%; specificity 53%. Per-level sensitivity 100%; specificity 73%
Tan et al., 2022, meta-analysis [64]	Systematic review/meta-analysis pooling 12 predominantly retrospective studies	1361 patients; 532,328 images used for model training	Pooled, predominantly per-image (retrospective)	Histopathology (per- included study)	Pooled sensitivity 90.3% (95% CI: 87.1–92.7%); specificity 84.4% (95% CI: 80.2–87.9%)

Table 4 presents the available AI-detection evidence at the level of individual study rather than as an indirect conventional-versus-AI comparison. Because these studies differ in design, setting, sample size, and unit of analysis, the rows should be read individually rather than pooled or ranked against a conventional-endoscopy comparator. Abbreviations: CI, confidence interval.

**Table 5:** Clinical function and evidence tier of Barrett esophagus diagnostic and surveillance technologies.

Technology	Primary Clinical Function	Evidence Tier	Key Caveat
WLE + Seattle-protocol biopsy	Confirmation/diagnosis; dysplasia detection (reference)	Established/guideline-recognized (reference standard)	Itself the comparator for all other tests; limited by sampling error [12]
High-definition endoscopy (HDE)	Confirmation/diagnosis; dysplasia detection	Established/guideline-recognized (minimum standard) [2,33]	Improves lesion detection but does not replace random biopsy [22]
Narrow-band imaging (NBI–BING)	Dysplasia/neoplasia detection and characterization	Guideline-recognized adjunct (PIVI-qualifying) [33]	Requires training; targeted-biopsy substitution applies only to regular NBI patterns [24,25]; used as an adjunct to, not a replacement for, a structured biopsy protocol [10]
Acetic acid chromoendoscopy (AAC)	Dysplasia/neoplasia detection	Guideline-recognized adjunct (PIVI-qualifying) [33]	Equipment-free but technique- and experience-dependent [26]; used as an adjunct to, not a replacement for, a structured biopsy protocol [10]
Confocal laser endomicroscopy (CLE)	Dysplasia/neoplasia characterization	Investigational	Cost, narrow field of view, access to training [28,29]
OCT/volumetric laser endomicroscopy (VLE)	Dysplasia characterization; post-ablation surveillance (buried-gland detection)	Investigational	Heterogeneous per-lesion evidence; not guideline-mandated [30,31,32]
p53 immunohistochemistry (diagnostic adjudication)	Diagnostic adjudication (indefinite-for-dysplasia)	Guideline-recognized adjunct [36]	Highly specific but too insensitive to use alone [35]
p53 immunohistochemistry (progression-risk marker)	Progression-risk stratification	No guideline recommendation	No guideline recommendation for or against routine use to predict progression to HGD/EAC, distinct from its established diagnostic-adjudication role [2,10]
DNA content/ploidy analysis	Progression-risk stratification	Investigational	Best-validated research marker but not incorporated into routine guideline pathways [37,38]
Multi-gene methylation panels	Progression-risk stratification	Investigational	Sensitivity near 50% in multicenter validation [40], lacks prospective validation
Cytosponge-TFF3	Screening/case-finding	Guideline-recognized (ACG conditional recommendation as a screening alternative) [2]	Requires prospective validation and generalizability to different health systems [42,43]; not established as a replacement for endoscopic surveillance in patients with known BE [2]
Cytosponge biomarker panel	Progression-risk stratification/triage in established BE	Investigational	AGA has acknowledged but not endorsed biomarker panels for progression-risk triage in established BE [10]
Esophageal capsule endoscopy (ECE)	Screening	Investigational	Cannot obtain tissue; cost-ineffective vs. EGD in decision-analytic models [52,53,54]
Breath testing (electronic nose)	Screening	Investigational	No standardized protocol; external validation still limited [55,56]
Circulating miRNA (EMERALD)	Screening (blood-based)	Investigational	Prospective validation pending [57]
AI-guided real-time CADe	Dysplasia/neoplasia detection	Investigational	Not yet incorporated into ACG/ASGE guidelines as a standard of care; limited external validation [60,61,62,63,64,65,66]. Emerging YOLO-based object-detection + hyperspectral-imaging pipelines extend this approach but remain investigational and are validated mainly in non-BE esophageal cancer cohorts [67,68]
AI-assisted Cytosponge/histology reading	Screening/confirmation support (pathology triage)	Investigational	Early-stage validation; not guideline-endorsed [70,71]

Evidence tiers reflect current society guidance (e.g., ACG [2], ASGE [15,33], BSG [36], and AGA [10]) and the ASGE PIVI framework where applicable, not the magnitude of a single reported sensitivity or specificity. Technology can be well-validated for one clinical function (e.g., p53 IHC in diagnostic adjudication) while lacking a guideline recommendation for another (e.g., p53 IHC in progression-risk stratification); functions and tiers are therefore assigned per use, not per technology in the abstract. Abbreviations: AI, artificial intelligence; BING, Barrett’s International NBI Group; CADe, computer-aided detection; CLE, confocal laser endomicroscopy; EGD, esophagogastroduodenoscopy; HDE, high-definition endoscopy; IHC, immunohistochemistry; miRNA, microRNA; NBI, narrow-band imaging; OCT, optical coherence tomography; PIVI, Preservation and Incorporation of Valuable Endoscopic Innovations; TFF3, trefoil factor 3; VLE, volumetric laser endomicroscopy; WLE, white-light endoscopy.

## Data Availability

No new data was generated or analyzed in this review. All data discussed are available in the tables and cited references.

## Abbreviations

The following abbreviations are used in the manuscript:

AAC	Acetic acid chromoendoscopy
AI	Artificial intelligence
ASGE	American Society for Gastrointestinal Endoscopy
AUROC	Area under the receiver-operating-characteristic curve
BE	Barrett’s esophagus
BEST2/BEST3	Barrett’s Esophagus Screening Trial 2/3
BING	Barrett’s International NBI Group
CI	Confidence interval
CLE	Confocal laser endomicroscopy
CNN	Convolutional neural network
DNA	Deoxyribonucleic acid
EAC	Esophageal adenocarcinoma
ECE	Esophageal capsule endoscopy
eCLE	Endoscope-integrated confocal laser endomicroscopy
EGD	Esophagogastroduodenoscopy
EMERALD	Esophageal MicroRNAs of BaRRett, Adenocarcinoma, and Dysplasia
e-nose	Electronic nose
FISH	Fluorescence in situ hybridization
GC-MS	Gas chromatography-mass spectrometry
GEJ	Gastroesophageal junction
GERD	Gastroesophageal reflux disease
H&E	Hematoxylin and eosin
HDE	High-definition endoscopy
HD-WLE	High-definition white-light endoscopy
HGD	High-grade dysplasia
IHC	Immunohistochemistry
LGD	Low-grade dysplasia
LOH	Loss of heterozygosity
miRNA	MicroRNA
NBI	Narrow-band imaging
NPV	Negative predictive value
OCT	Optical coherence tomography
OR	Odds ratio
pCLE	Probe-based confocal laser endomicroscopy
PIVI	Preservation and Incorporation of Valuable Endoscopic Innovations
PPV	Positive predictive value
QALY	Quality-adjusted life-year
RCT	Randomized controlled trial
SD-WLE	Standard-definition white-light endoscopy
TFF3	Trefoil factor 3
VLE	Volumetric laser endomicroscopy
VOC	Volatile organic compound
WLE	White-light endoscopy
